# AO Spine-DGOU Osteoporotic Fracture Classification System: Internal Validation by the AO Spine Knowledge Forum Trauma

**DOI:** 10.1177/21925682241288187

**Published:** 2024-09-27

**Authors:** Julian Scherer, Andrei Joaquim, Alex Vaccaro, Rishi Kanna, Mohammad El-Sharkawi, Masahiko Takahata, Mohamed M. Aly, Gaston Camino-Willhuber, Ulrich Spiegl, Cumhur Oner, Jose A. Canseco, Ratko Yurac, Lorin Michael Benneker, Eugen Cezar Popescu, Richard Bransford, Harvinder Singh Chhabra, Frank Kandziora, Marko H. Neva, Klaus John Schnake

**Affiliations:** 1 General Medicine & Global Health (GMGH), Department of Medicine and Orthopaedic Research Unit (ORU), Division of Orthopaedic Surgery, Faculty of Health Sciences, University of Cape Town, South Africa; 2 Department of Traumatology, 27217 University Hospital of Zurich, Zürich, Switzerland; 3 Neurosurgery Division, Department of Neurology, 28132 State University of Campinas, Campinas-Sao Paulo, Brazil; 4 Department of Orthopaedic Surgery, Rothman Institute, Thomas Jefferson University Hospital, Philadelphia, PA, USA; 5 Spine Department of Orthopaedics and Spine Surgery, 76290 Ganga Hospital, Coimbatore, India; 6 Department of Orthopaedic and Trauma Surgery, Faculty of Medicine, 68796 Assiut University, Assiut, Egypt; 7 Department of Orthopaedic Surgery, Dokkyo Medical University School of Medicine, Soka, Japan; 8 Department of Neurosurgery, Prince Mohammed Bin Abdulaziz Hospital, Riyadh, Saudi Arabi; 9 Department of Neurosurgery, Mansoura University, Mansoura, Egypt; 10 Department of Traumatology, Policina Gipuzkoa, San Sebastian, Spain; 11 Klinik für Unfallchirurgie und Orthopädie, 14953 Klinik München Harlaching, München, Germany; 12 Department of Orthopaedics, 8124 University Medical Centers, Utrecht, the Netherlands; 13 Clinica Alemana de Santiago, 441184 University Del Desarrollo, Vitacura, Chile; 14 Department for Spine Surgery, Sonnenhof Spital, University of Bern, Switzerland; 15 Department of Neurosurgery, “Prof. N. Oblu” Emergency Hospital, Iasi, Romania; 16 Department of Orthopaedics and Sports Medicine, 21618 Harborview Medical Center, University of Washington, Seattle, WA, USA; 17 Sri Balaji Action Medical Institute, New Delhi, India; 18 Center for Spine Surgery and Neurotraumatology, BG Unfallklinik Frankfurt Am Main, Frankfurt, Germany; 19 Department of Orthopaedic and Trauma Surgery, 60670 Tampere University Hospital, Tampere, Finland; 20 Center for Spinal and Scoliosis Surgery, 232691 Malteser Waldkrankenhaus St. Marien Erlangen, Erlangen, Germany; 21 Department of Orthopedics and Traumatology, Paracelsus Private Medical University Nuremberg, Nuremberg, Germany

**Keywords:** osteoporosis, spinal fracture, fracture classification, validation study

## Abstract

**Study Design:**

Cross-sectional survey.

**Objectives:**

Injury classifications are important tools to identify fracture patterns, guide treatment-decisions and aid to identify optimal treatment plans. The AO Spine-DGOU Osteoporotic Fracture (OF) classification system was developed, and the aim of this study was to assess the reliability of this new classification system.

**Methods:**

23 Members of the AO Spine Knowledge Forum Trauma participated in the validation process. Participants were asked to rate 33 cases according to the OF classification at 2 time points, 4 weeks apart (assessment 1 and 2). The kappa statistic (κ) was calculated to assess inter-observer reliability and intra-rater reproducibility. The gold master key for each case was determined by approval of at least 5 out of 7 members of the DGOU.

**Results:**

A total of 1386 ratings (21 raters) were performed. The overall inter-rater agreement was moderate with a combined kappa statistic for the OF classification of 0.496 in assessment 1 and 0.482 in assessment 2. The combined percentage of correct ratings (compared to gold-standard) in assessment 1 was 71.4% and 67.4% in assessment 2. The average intra-rater reproducibility was substantial (κ = 0.74, median 0.76, range 0.55 to 1.00, SD 0.13) for the assessed fracture types.

**Conclusions:**

The assessed overall inter-rater reliability was moderate and substantial in some instances. The average intra-rater reproducibility is substantial. It seems that appropriate training of the classification system can enhance inter- and intra-rater reliability.

## Background

Osteoporosis represents a major public health concern with the elderly population steadily rising worldwide.^1^ Vertebral fractures are the most common pathological fractures as a result of low bone mass and bone fragility due to osteoporosis^2,3^ leading to morbidity and potential mortality.^4^ The management of osteoporotic vertebral fractures (OVFs) can be challenging due to patients’ age, existing comorbidities, reduced physiological reserves and often multipharmacy.^5^ Osteoporotic changes in the elderly can either be a contributing factor in traumatic (low-energy) vertebral fractures or can be the cause of those fractures.^4^ Injury classifications are important tools for patient care as well as for communication amongst caregivers. Classification systems, ultimately, should guide physicians in decision-making for optimal treatment of the patient. Therefore, classification systems should be simple, reproducible and should accentuate important fracture characteristics which are relevant for patient care. Commonly used and internationally accepted classifications for (thoracolumbar) vertebral fractures were not specifically developed for patients with osteoporotic changes. This differentiation is important since OVFs behave and are managed differently compared to traumatic (high-energy) vertebral fractures.^6^

Recently, the Spine Section of the German Society for Orthopaedics and Trauma (DGOU), who have formed the working group “Osteoporotic Fractures”, have proposed a new classification for osteoporotic thoraco-lumbar spine fractures.^4^

The final AO Spine-DGOU Osteoporotic Fracture (OF) classification system was developed after several consensus meetings with members the AO Spine Knowledge Forum Trauma. Blattert et al proposed treatment recommendations based on the OF classification: OF 1 and 2 should be treated conservatively, whereas OF 3 can either be managed conservatively or surgically. OF 4 and OF 5 are unstable fractures which should be treated surgically.^7^ A large multi-center study from Germany, assessing performed treatment strategies of OVFs, showed a high accordance with the OF-based treatment recommendations.^6^ Whereas treatment recommendations were assessed and validated with existing clinical data, data on the reliability of the AO Spine-DGOU OF classification is lacking.

Thus, the aim of the present study was to assess the reliability of the AO Spine-DGOU OF classification amongst members of the AO Spine Knowledge Forum Trauma.

## Material and Methods

### Development of Classification System

The methodology behind the creation of the AO Spine-DGOU classification has been described in detail in the publication of the Spine Section of the German Society for Orthopaedics and Trauma (DGOU).^4^

### The AO Spine-DGOU Osteoporotic Fracture (OF) Classification

This classification consists of 5 groups: OF 1, no deformation, only bone edema assessed with MRI, OF 2, deformation of 1 endplate with no or minor involvement of posterior wall, OF 3, deformation of 1 endplate with substantial involvement of posterior wall, OF 4, deformation of both endplates with or without involvement of posterior wall, and OF 5, fracture with anterior or posterior tension band failure. (Supplement 1)

### Validation Process

Members of the AO Spine Knowledge Forum Trauma, an international group of spinal surgeons dedicated to the study of spinal trauma from different regions of the world, participated in the validation process. Participants were asked to rate cases according to the OF classification at 2 time points, 4 weeks apart (assessment 1 and 2), to avoid recall bias. Participants were not particularly familiar with the classification but an introduction to the classification with example cases was given and participating surgeons were able to use the classification poster for the assessments and were asked to provide any feedback they may have to improve the classification and validation process. All of the selected cases included osteoporotic thoracolumbar fractures with different morphological subtypes. A total of 33 cases were selected, and 7 DGOU members developed the gold-standard master key for each case. In cases of disagreement, consensus was reached. All cases were reviewed independently by 23 surgeons from the AO Spine Knowledge Forum Trauma representing different regions of the world. To support the surgeons in rating the cases, key-images showing the fracture, and short video sequences of computed tomography and/or MRI were provided. Cases were randomized in both assessments.

### Statistical Analysis

The kappa statistic (κ) was calculated to assess the reliability of the classification system among different observers (inter-rater agreement) and the reproducibility for the same observer on separate occasions (intra-rater reproducibility).^8^ The Fleiss kappa statistic measures the agreement of multiple raters who rate multiple subjects, with the rating based on multiple categories. The coefficients were interpreted utilizing the Landis and Koch grading system, which defines κ ≤ 0.2 as slight reliability (agreement/reproducibility), 0.2 < κ ≤ 0.4 as fair reliability, 0.4 < κ ≤ 0.6 as moderate reliability, 0.6 < κ ≤ 0.8 as substantial reliability, and κ > 0.8 as excellent reliability.^9^ Inter-rater and intra-rater agreement was calculated for the different classification groups (OF 1-5) and overall, for the classification itself. Furthermore, agreement with the gold-standard (accuracy) was calculated overall and for each OF-subtype.

### Development of the OF Classification System

**Figure 1.**
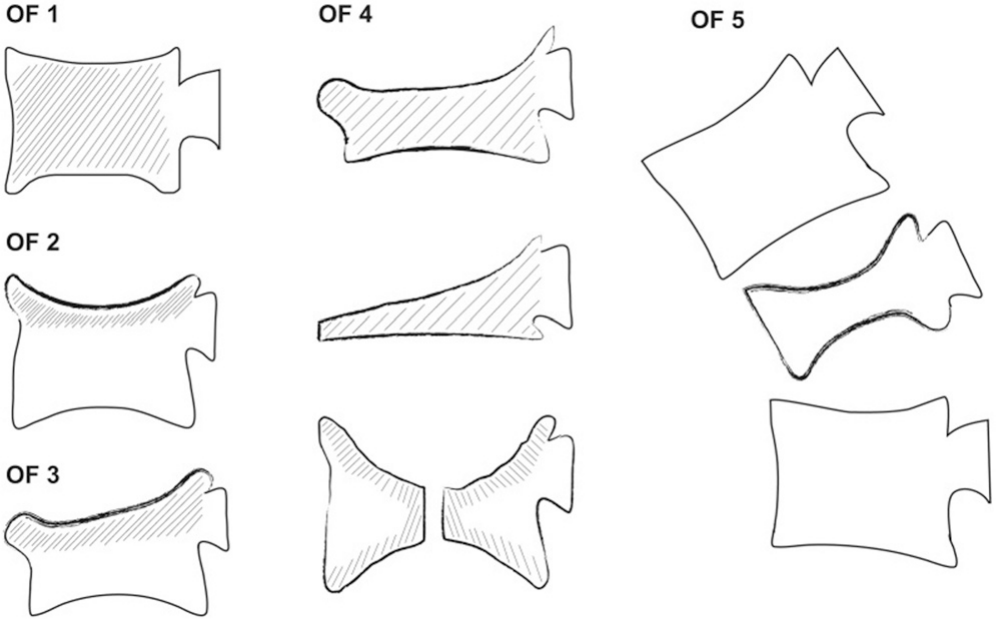
Development of the OF classification system. OF 1: No vertebral deformation (vertebral body edema in MRI-STIR only). X-rays do not show vertebral deformation. OF 2: Deformation of 1 endplate without or with only minor posterior wall involvement (<1/5). OF 3: Deformation of 1 endplate with distinct posterior wall involvement (>1/5). OF 4: Deformation of both endplates with or without posterior wall involvement. This type can lead to the loss of integrity of the vertebral frame structure (complete burst), or vertebral body collapse, or pincer-type fracture. OF 5: Injuries with anterior or posterior tension band failure. These injuries have signs of distraction, rotation, or translation.

The classification consists of 5 different subtypes (OF 1-5)^4^ (Figure 1).

## Results

Out of 23 raters, 2 were excluded from final analysis due to non-compliance in the assessment process. A total of 1386 (693 twice) ratings were performed. Of the 33 presented cases, 1 case was classified (gold-standard) as OF 1 (3%), ten as OF 2 (30.3%), 5 as OF 3 (15.1%), eleven as OF 4 (33.3%) and 6 as OF 5 (18.2%).

### Inter-Rater Agreement

The overall inter-rater agreement was moderate with a combined kappa statistic for the OF classification of 0.496 in assessment 1 and 0.482 in assessment 2, respectively. The lowest level of agreement was seen in OF 1 (κ = 0.293, 0.181 in assessment 2), whereas the highest level of agreement was observed in OF 4 (κ = 0.625, 0.683 respectively). (Table 1) In both assessments, the inter-rater levels of agreement for OF 2, 4 and 5 were higher than the overall level of agreement.

**Table 1.** Inter-rater Agreement of the OF Classification.

Classification	Assessment 1 (κ)	Assessment 2 (κ)
Total	0.496	0.482
OF 1	0.293	0.181
OF 2	0.576	0.579
OF 3	0.469	0.363
OF 4	0.625	0.683
OF 5	0.518	0.603

### Agreement with Gold-Standard

The combined percentage of correct ratings (compared to gold-standard) in assessment 1 was 71.4% and 67.4% in assessment 2. (Table 2) In both assessments, the lowest level of agreement with the gold standard was seen in OF 1 and OF 5 fractures (61.9% and 38.1%, 59.5% and 67.5% respectively). Half of the raters assessed the OF 1 (1 case in each assessment) falsely as an OF 2. One of the 6 as OF 5 classified cases were rated wrongly by most of the raters (59.5%). The overall mean agreement with the gold-standard among raters in this study was 75.04% (median 75.76%, range 63.64% to 84.85%, SD 6.28) in assessment 1 and 75.90% (median 78.79%, range 57.58% to 84.85%, SD 8.65).

**Table 2.** Agreement With Gold-Standard.

Classification	Assessment 1 (%)	Assessment 2 (%)
Combined	71.4	67.4
OF 1	61.9	38.1
OF 2	76.2	79.0
OF 3	76.2	67.6
OF 4	83.1	84.8
OF 5	59.5	67.5

### Intra-Rater Agreement

The average intra-rater reproducibility was substantial (κ = 0.74, median 0.76, range 0.55 to 1.00, SD 0.13) for the assessed fracture types. Three of the reviewers showed a moderate level of agreement (0.4 < κ ≤ 0.6), whereas twelve raters had a substantial level of agreement (0.6 < κ ≤ 0.8) and 6 of the reviewers were assessed with an excellent level of agreement (κ > 0.8).

## Discussion

The prevalence of OVFs varies between approximately 19% in women and 20% in men amongst the elderly population.^10^ Due to a rising number of elderly people worldwide, the incidence rates of these fractures can be assumed to rise. OVFs represent a major public health concern due to increased morbidity and mortality, especially in patients with an age of 75 years and above.^11^ Accurate diagnosis and treatment of these fractures is crucial to avoid prolonged and chronic episodes of pain, to reduce morbidity and mortality and to prevent kyphosis and further collapse of the fractured vertebra which can result in neurological deficits. However, to date, there is no consensus guideline on the treatment of OVFs and conservative treatment is considered the primary treatment option.^12^ Existing classification systems on thoracolumbar vertebral fractures such as the AO Spine thoracolumbar spine injury classification system or the TLICS are widely accepted but are unsuitable for OVFs since these systems are not appreciating the distinct features of vertebral fractures with underlying osteoporotic changes.^13,14^ Recently, a new classification specifically addressing OVFs was introduced by the working group on “Osteoporotic Fractures” of the DGOU and was then further developed in cooperation with AO Spine (AO Spine-DGOU Osteoporotic Fracture (OF) classification system) which grades OVFs into 5 groups and gives treatment recommendations (surgical vs conservative) for each classification group.^4^ Thus, the aim of this study was to assess the inter-rater as well as intra-rater reliability amongst AO Spine Knowledge Forum Trauma members.

We assessed a moderate combined overall inter-rater agreement in both assessments (κ = 0.496 in assessment 1 and κ = 0.482 in assessment 2) which is in keeping with 2 previous studies assessing the inter-rater agreement of this classification amongst 6 surgeons in each study.^15,16^ Furthermore, we found that our findings are comparable to the previous international validation study of the AO Spine thoracolumbar injury classification system (κ = 0.56).^17^ Due to global osteoporotic changes of the vertebrae on the assessed imaging data, rating of the fractures might be more difficult (eg, degenerative endplate changes vs traumatic endplate changes) and therefore, the assessed inter-rater agreement might be falsely low. Interestingly, the inter-rater agreement declined between the first and the second assessment. OF 1 showed fair agreement (κ = 0.293) in assessment 1 and slight agreement (κ = 0.181) which represents the most inferior inter-rater agreement amongst the 5 classification groups. This relative low agreement rate may be also explained by the low prevalence of this morphology in the case sample as well as the difficulty to distinguish degenerative endplate irregularities from traumatic changes. OF 4 and OF 5 are recommended for surgical treatment and showed a substantial inter-rater agreement in assessment 2, which highlights the uniformity of rating and possibly treatment decisions.^7^ The assessed moderate overall inter-rater agreement could be further due to lack of training of the included raters, which were all experienced raters with good training in well-established classifications who did not undergo training of the AO Spine-DGOU Osteoporotic Fracture (OF) classification system prior this assessment, which highlights the need for appropriate training of classification systems. This should be assessed further after appropriate training sessions of the respected classification system. We observed a combined percentage of correct ratings, compared to gold-standard, of 71.4% in assessment 1 and 67.4% in assessment 2. Like the inter-rater agreement, OF 1 was rated incorrectly the most often in both assessments and was rated worse in assessment 2 (61.9% and 38.1%). Half of the raters rated OF 1 as OF 2. This could be due to over-interpretation of MRI imaging ie, interpreting physiological endplate deformities with bone marrow edema as impression fracture. Therefore, assessment of both, CT and MRI are necessary to avoid an over-interpretation and to increase inter-rater and agreement with the gold-standard, which has been also shown in a previous assessment of 6 surgeons.^16,18^ According to the newly introduced OF-score, both fracture types, in the worst clinical scenario, could be treated surgically.^7^ However, a multi-center study showed that OF 1 are almost always and OF 2 are commonly treated conservatively.^6^ A possible second solution to increase the intra-rater agreement as well as the agreement with the gold-standard would therefore be to fuse OF 1 and OF 2 into 1 entity and make surgical decision-making solely dependent on the modifiers according to the OF-score.

We assessed a substantial overall intra-rater reproducibility of 0.74 which is comparable to the AO Spine thoracolumbar spine injury classification system (κ = 0.68). This finding is also comparable to the assessment from Germany, which found a substantial intra-observer agreement when raters were provided with radiograph and MRI (κ = 0.64). This study, in addition, showed that the intra-observer reliability can be increased by providing CT-scans as an additional imaging modality, which highlights the importance of complete imaging.^16,17^ The majority of the assessed raters showed a substantial or excellent reproducibility.

This study has certain limitations. Firstly, there might be a varying degree of familiarity with the OF-classification amongst the raters which results in a classification knowledge-inequality and could result in a potential bias. Furthermore, the sole use of key-images (in some cases) or vice versa might have an impact on the rater`s response. It is likely that some of the reviewers would change their response while assessing a complete imaging file rather than solely key images. Another potential limitation is the inequal frequency of different fracture subtypes and hence, the data on rare subtypes (eg, OF 1) should be interpreted with caution. Additionality, some of the presented images showed multiple fractures which might have caused confusion regarding the fracture to rate, decreasing the reliability rate. Furthermore, the members of the AO Spine Knowledge Forum Trauma might have varying degrees of knowledge on interpreting different imaging modalities and familiarity with the presented classification since not all members were involved in the development process, which could result in an inhomogeneous group of raters and could have a negative effect on the inter-rater agreement. Standardized training on imaging and of the OF-classification could limit these potential biases.

## Conclusion

Like previously described AO Spine thoracolumbar fracture classifications as well as small inter- and intra-rater reliability assessments, the results from the present study showed moderate to substantial interrater reliability and substantial inter-rater reproducibility amongst a group of members of the AO Spine Knowledge Forum Trauma. As a next step, an international validation amongst spine surgeons with a higher number of raters representing different backgrounds, work settings, and regions should be performed to assess the AO Spine-DGOU OF classification reliability globally. It seems that appropriate training of the respected classification system is necessary to increase inter- and intra-rater reliability.

## Supplemental Material

Supplemental Material - AO Spine-DGOU Osteoporotic Fracture (OF) Classification System: Internal Validation by the AO Spine Knowledge Forum Trauma Supplemental Material for AO Spine-DGOU Osteoporotic Fracture (OF) Classification System: Internal Validation by the AO Spine Knowledge Forum Trauma by Julian Scherer, Andrei Joaquim, Alex Vaccaro, Rishi Kanna, Mohammad El-Sharkawi, Masahiko Takahata, Mohamed M. Aly, Gaston Camino-Willhuber, Ulrich Spiegl, Cumhur Oner, Jose A. Canseco, Ratko Yurac, Lorin Michael Benneker, Eugen Cezar Popescu, Richard Bransford, Chhabra Harvinder Singh, Frank Kandziora, Marko H. Neva, and Klaus John Schnake in Global Spine Journal

## Data Availability

Data is available upon reasonable request towards the corresponding author.

## ORCID iDs

Julian Scherer https://orcid.org/0000-0001-8943-5817

Andrei Joaquim https://orcid.org/0000-0003-2645-0483

Rishi Kanna https://orcid.org/0000-0001-5817-4909

Mohammad El-Sharkawi https://orcid.org/0000-0001-6177-7145

Mohamed M. Aly https://orcid.org/0000-0001-5221-8288

Gaston Camino-Willhuber https://orcid.org/0000-0002-5684-7679

Ulrich Spiegl https://orcid.org/0000-0002-5179-4192

Jose A. Canseco https://orcid.org/0000-0002-2152-5725

Ratko Yurac https://orcid.org/0000-0003-3603-6294

Eugen Cezar Popescu https://orcid.org/0000-0001-5732-5402

## References

[1] Reginster JY Burlet N . Osteoporosis: a still increasing prevalence. Bone. 2006;38(2 Suppl 1):S4-9. 16455317 10.1016/j.bone.2005.11.024

[2] Ensrud KE . Epidemiology of fracture risk with advancing age. J Gerontol A Biol Sci Med Sci. 2013;68(10):1236-1242. 23833201 10.1093/gerona/glt092

[3] Khavinson V Popovich I Mikhailova O . Towards realization of longer life. Acta Biomed. 2020;91(3):e2020054. 32921699 10.23750/abm.v91i3.10079 PMC7716987

[4] Schnake KJ Blattert TR Hahn P , et al. Classification of osteoporotic thoracolumbar spine fractures: recommendations of the spine section of the German society for Orthopaedics and trauma (DGOU). Global Spine J. 2018;8(2 Suppl):46s-49s. 30210960 10.1177/2192568217717972 PMC6130101

[5] Hadji P Klein S Gothe H , et al. The epidemiology of osteoporosis--Bone Evaluation Study (BEST): an analysis of routine health insurance data. Dtsch Arztebl Int. 2013;110(4):52-57. 23413388 10.3238/arztebl.2013.0052 PMC3570954

[6] Ullrich BW Schenk P Scheyerer MJ , et al. Georg Schmorl prize of the German spine society (DWG) 2022: current treatment for inpatients with osteoporotic thoracolumbar fractures—results of the EOFTT study. Eur Spine J. 2023;32(5):1525-1535. 36595136 10.1007/s00586-022-07519-x

[7] Blattert TR Schnake KJ Gonschorek O , et al. Nonsurgical and surgical management of osteoporotic vertebral body fractures: recommendations of the spine section of the German Society for Orthopaedics and Trauma (DGOU). Global Spine J. 2018;8(2_suppl):50S-55S. 30210962 10.1177/2192568217745823 PMC6130106

[8] Fleiss JL . Measuring nominal scale agreement among many raters. Psychol Bull. 1971;76(5):378-382.

[9] Landis JR Koch GG . The measurement of observer agreement for categorical data. Biometrics. 1977;33(1):159-174. 843571

[10] Waterloo S Ahmed LA Center JR , et al. Prevalence of vertebral fractures in women and men in the population-based Tromsø Study. BMC Muscoskel Disord. 2012;13:3. 10.1186/1471-2474-13-3 PMC3273434 22251875

[11] Cauley JA . Public health impact of osteoporosis. J Gerontol A Biol Sci Med Sci. 2013;68(10):1243-1251. 23902935 10.1093/gerona/glt093 PMC3779634

[12] Jang HD Kim EH Lee JC Choi SW Kim K Shin BJ . Current concepts in the management of osteoporotic vertebral fractures: a narrative review. Asian Spine J. 2020;14(6):898-909. 33373513 10.31616/asj.2020.0594 PMC7788360

[13] Vaccaro AR Oner C Kepler CK , et al. AOSpine thoracolumbar spine injury classification system: fracture description, neurological status, and key modifiers. Spine. 2013;38(23):2028-2037. 23970107 10.1097/BRS.0b013e3182a8a381

[14] Vaccaro AR Lehman RA Jr. Hurlbert RJ , et al. A new classification of thoracolumbar injuries: the importance of injury morphology, the integrity of the posterior ligamentous complex, and neurologic status. Spine. 2005;30(20):2325-2333. 16227897 10.1097/01.brs.0000182986.43345.cb

[15] Quinteros G Cabrera JP Urrutia J , et al. Reliability evaluation of the new AO spine-DGOU classification for osteoporotic thoracolumbar fractures. World Neurosurg. 2022;161:e436-e440. 35158101 10.1016/j.wneu.2022.02.029

[16] Schonrogge M Lahodski V Otto R , et al. Inter- and intraobserver reliabilities and critical analysis of the osteoporotic fracture classification of osteoporotic vertebral body fractures. Eur Spine J. 2022;31(9):2431-2438. 35378632 10.1007/s00586-022-07201-2

[17] Kepler CK Vaccaro AR Koerner JD , et al. Reliability analysis of the AOSpine thoracolumbar spine injury classification system by a worldwide group of naïve spinal surgeons. Eur Spine J. 2016;25(4):1082-1086. 25599849 10.1007/s00586-015-3765-9

[18] Spiegl UJ Behr L Osterhoff G Rupprecht G Scheyerer MJ Katscher S . OF spine classification of osteoporotic thoracolumbar vertebral body fractures by MRI and conventional radiographs only leads to high inter-observer agreement rates-an additional CT adds limited information for the of classification and the OF score. BMC Muscoskel Disord. 2022;23(1):1086. 10.1186/s12891-022-06056-4 PMC9743708 36510215

