# Supplementary material for: AO Spine-DGOU Osteoporotic Fracture Classification System: Internal Validation by the AO Spine Knowledge Forum Trauma
Source: Global Spine J. 2024 Sep 27;15(4):2152–7. doi: 10.1177/21925682241288187 (PMC11559773; doi:10.1177/21925682241288187)
Supplement: Supplemental Material - AO Spine-DGOU Osteoporotic Fracture (OF) Classification System: Internal Validation by the AO Spine Knowledge Forum Trauma [file sj-pdf-1-gsj-10.1177_21925682241288187.pdf]

AO Spine—DGOU Osteoporotic Fracture (OF) Classification System

AO SPINE

OF 1

No deformation (vertebral body edema in MRI-STIR)

- Typically not visible on x-rays: chance to find on MRI

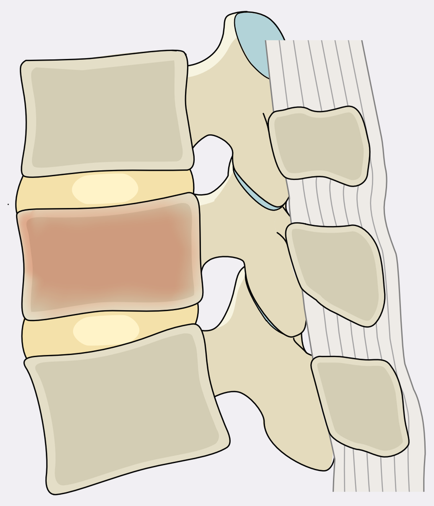

OF 2

Deformation of one endplate without or with only minor posterior wall involvement

- With posterior wall < 1/5 involvement

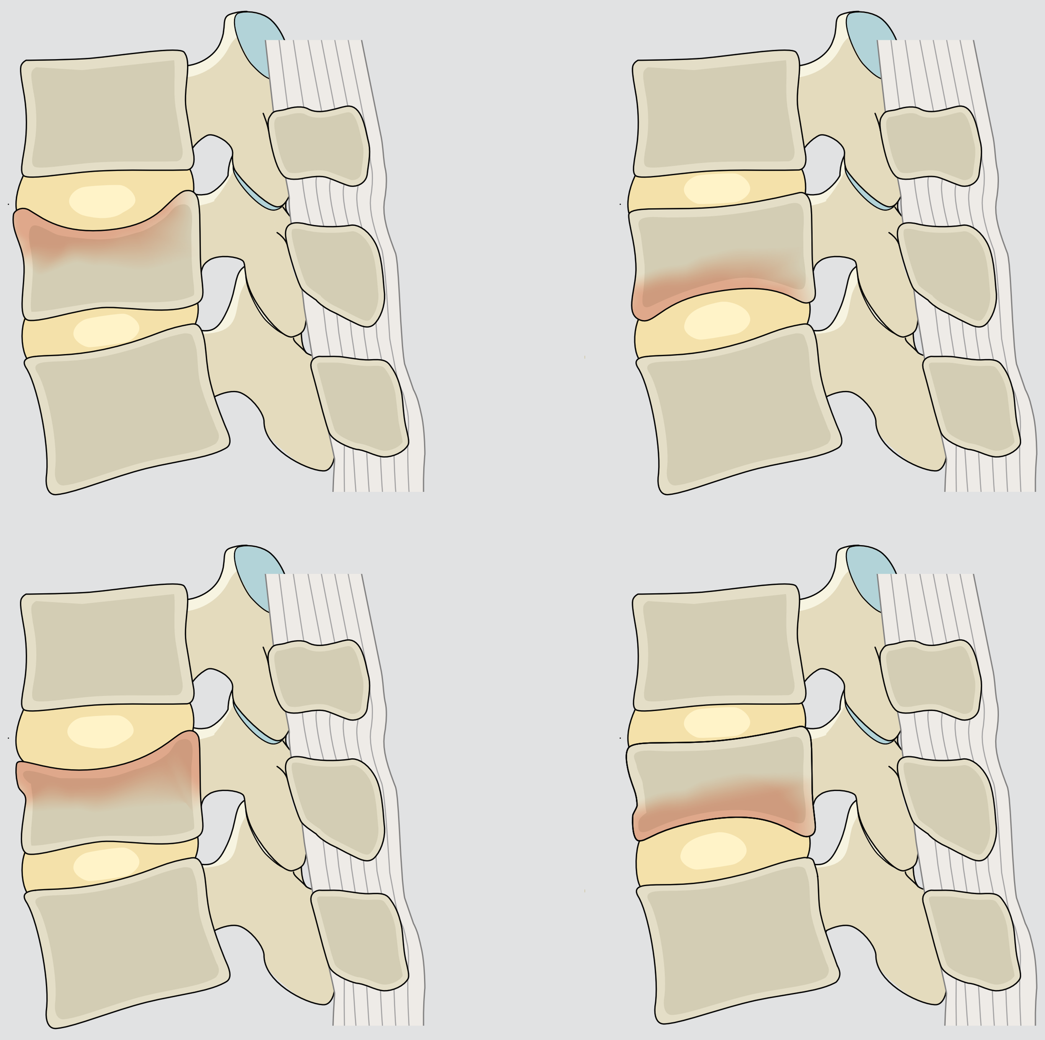

OF 3

Deformation of one endplate with distinct posterior wall involvement

- With posterior wall > 1/5 involved

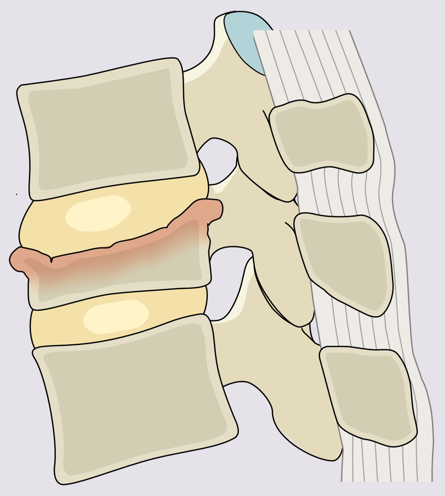

OF 4

Deformation of both endplates with/without posterior wall involvement

- Loss of vertebral frame structure
- Vertebral body collapse
- Pincer type fracture

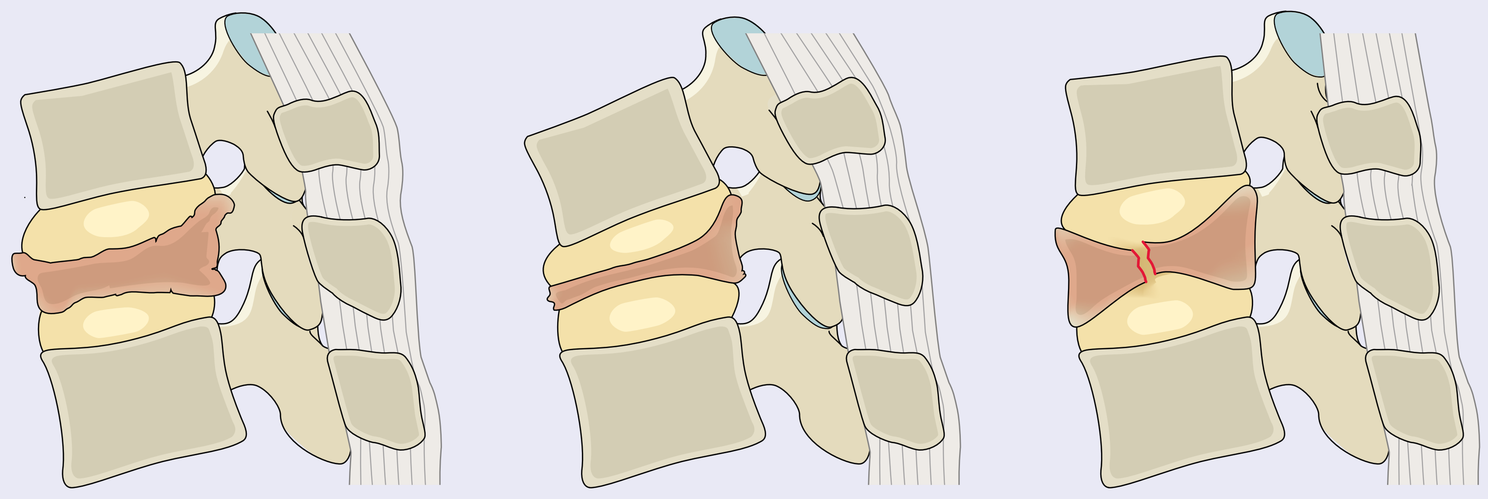

OF 5

Injuries with anterior or posterior tension band failure

- Injuries with signs of distraction, rotation, or translation
- Hyperextension with anterior tension band failure

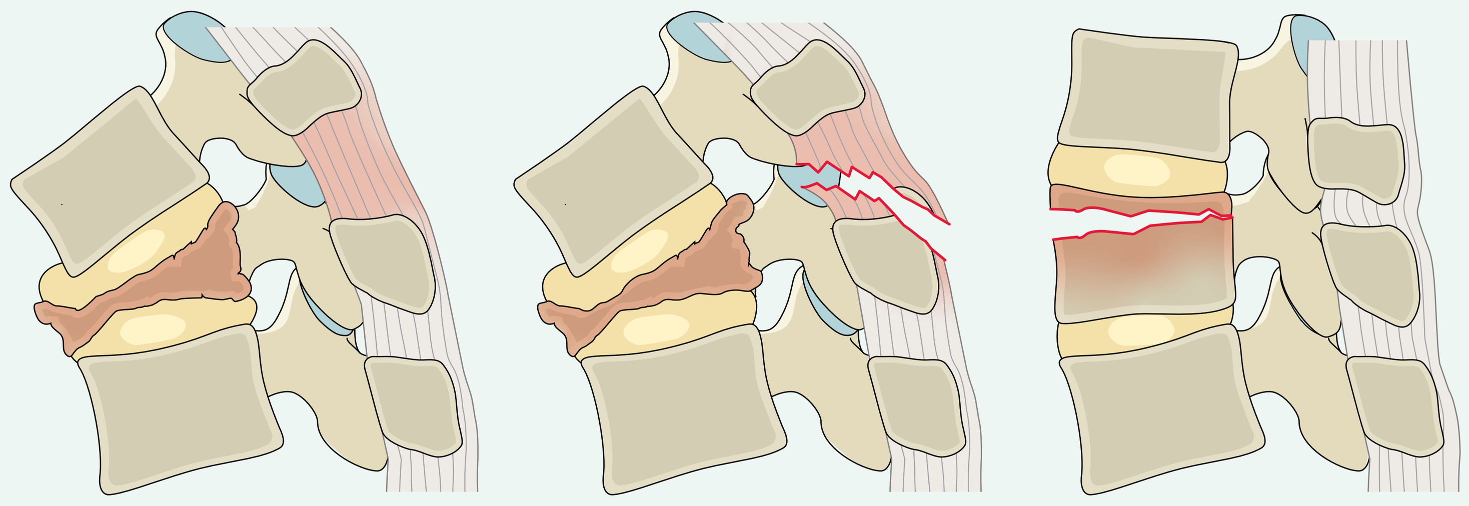

Algorithm for morphologic classification

START → Failure of anterior/posterior tension band? → YES → **OF 5**

NO ↓

Deformity of both endplates with/without posterior wall involvement? → YES → **OF 4**

NO ↓

Deformity of one endplate? → YES → Distinct posterior wall involvement? → YES → **OF 3**

Distinct posterior wall involvement? → NO → **OF 2**

Deformity of one endplate? → NO ↓

Vertebral body edema? → YES → **OF 1**

Vertebral body edema? → NO → **No injury**

Modified score for therapeutic decision making in OF*

Parameter	Grade	Points
Morphology (OF 1-5)	1-5	2-10
Severity of Osteoporosis	T-Score <-3 or qCT: HU ≤90	1
Deformity Progression	Yes, No	1, -1
Pain (under analgesia)**	VAS ≥5, <5	1, -1
Neurological Symptoms (N2-N4)	Yes	2
Mobilisation (under analgesia)	No, Yes	1, -1
Health Status	ASA >3, ***mFI >2, Anticoagulation	Each -1, Maximum -2

0 points if a parameter is unknown or not determinable;
0–5 points = Conservative therapy;
6 points = Conservative therapy or surgery;
> 6 points = Surgery.

* The severity score system has not been validated yet and should be used as a reference only.
** According to step II WHO pain ladder.
*** 5-item modified frailty index (mFI) = COPD, or recent pneumonia; Congestive heart failure; Functional status (not independent); Hypertension requiring medication; Diabetes mellitus.

© 2020 AO Foundation, AO Spine
This work is licensed under the Creative Commons Attribution-NonCommercial-NoDerivatives 4.0 International License.
To view a copy of this license, visit http://creativecommons.org/licenses/by-nc-nd/4.0/

Further information:
www.aospine.org/classification
